# Septin-2 is overexpressed in epithelial ovarian cancer and mediates proliferation via regulation of cellular metabolic proteins

**DOI:** 10.18632/oncotarget.26836

**Published:** 2019-04-26

**Authors:** Nicole E. James, Evelyn Cantillo, Naohiro Yano, Clinton O. Chichester, Paul A. DiSilvestro, Virginia Hovanesian, R. Shyama Prasad Rao, Kyukwang K. Kim, Richard G. Moore, Nagib Ahsan, Jennifer R. Ribeiro

**Affiliations:** ^1^Division of Gynecologic Oncology, Program in Women’s Oncology, Department of Obstetrics and Gynecology, Women and Infants Hospital, Providence, RI, USA; ^2^Department of Biomedical and Pharmaceutical Sciences, University of Rhode Island, Kingston, RI, USA; ^3^Department of Surgery, Roger Williams Medical Center, Boston University Medical School, Providence, RI, USA; ^4^Core Research Laboratories, Rhode Island Hospital, Providence, RI, USA; ^5^Biostatistics and Bioinformatics Division, Yenepoya Research Center, Yenepoya University, Mangalore, India; ^6^Division of Gynecologic Oncology, Department of Obstetrics and Gynecology, Wilmot Cancer Institute, University of Rochester Medical Center, Rochester, NY, USA; ^7^Center for Cancer Research Development, Proteomics Core Facility, Rhode Island Hospital, Providence, RI, USA; ^8^Division of Biology and Medicine, Warren Alpert Medical School, Brown University, Providence, RI, USA

**Keywords:** ovarian cancer, proteomics, septin-2, metabolic proteins

## Abstract

Epithelial Ovarian Cancer (EOC) is associated with dismal survival rates due to the fact that patients are frequently diagnosed at an advanced stage and eventually become resistant to traditional chemotherapeutics. Hence, there is a crucial need for new and innovative therapies. Septin-2, a member of the septin family of GTP binding proteins, has been characterized in EOC for the first time and represents a potential future target. Septin-2 was found to be overexpressed in serous and clear cell human patient tissue compared to benign disease. Stable septin-2 knockdown clones developed in an ovarian cancer cell line exhibited a significant decrease in proliferation rates. Comparative label-free proteomic analysis of septin-2 knockdown cells revealed differential protein expression of pathways associated with the TCA cycle, acetyl CoA, proteasome and spliceosome. Further validation of target proteins indicated that septin-2 plays a predominant role in post-transcriptional and translational modifications as well as cellular metabolism, and suggested the potential novel role of septin-2 in promoting EOC tumorigenesis through these mechanisms.

## INTRODUCTION

Epithelial Ovarian Cancer (EOC) is the most lethal gynecologic malignancy [1]. In 2018, there will be an estimated 22,240 new cases of EOC diagnosed and 14,070 deaths in the United States. While EOC accounts for only 2.5 % of all female cancers, it is responsible for 5% of all cancer deaths due to low disease survival rates [2]. These dire statistics are attributed to the fact that the majority of patients are diagnosed at an advanced stage. In addition, while patients generally respond well to frontline platinum-based chemotherapy, chemoresistant recurrences are common [3]. Therefore, there is a strong need for novel early detection methods and targeted therapies for EOC patients.

Septin-2 is a member of the septin family, a conserved family comprised of 13 GTP binding proteins [4]. Septins, which are structurally observed as rods and filaments, are vital to a number of cellular processes, including cytokinesis, vesicle trafficking, and exocytosis [5]. They are considered to be a fourth component of the cytoskeleton due to their association with actin, microtubules, and membranes [6]. Septins have been identified as having a role in neurodegenerative disease, since they were detected in brain tissue from patients with Alzheimer disease [7]. In addition, they have been reported to be involved in bacterial infections, Parkinson’s disease, and male infertility [8].

In more recent years, emphasis has been placed on investigating the role of septins in tumorigenesis [9]. Due to their natural function in scaffolding and membrane compartmentalization, it is plausible that they could also play a role in the organization of membrane associated proteins involved in diverse tumorigenic signaling pathways [6]. Septin-9 is the most well studied septin family member in relationship to cancer, and its methylation status is utilized as a biomarker in colorectal cancer [10]. However, there have also been numerous studies linking septin-2 to neoplasia. Thus far, septin-2 has been specifically implicated in Hodgkin’s lymphoma and biliary tract, gastric, hepatocellular, renal cell carcinoma, and breast cancer [11–17]. However, septin-2’s role in EOC has not been previously investigated.

In this study, we begin to elucidate septin-2’s function in EOC. As septins have been shown to have diverse roles in tumorigenesis, this is the first step in specifically defining septin-2’s contribution to EOC pathogenesis. To establish the clinical relevance of septin-2 in EOC, we first sought to compare levels of septin-2 in various histological pathologies of EOC versus benign disease. Furthermore, we present for the first time a global analysis of septin-2 mediated proteomics in EOC and describe signaling pathways most affected by septin-2 depletion. The results from this study lay the framework for future mechanistic studies to determine the precise role of septin-2 in EOC.

## RESULTS

### Septin-2 is overexpressed in EOC

**Figure 1:**
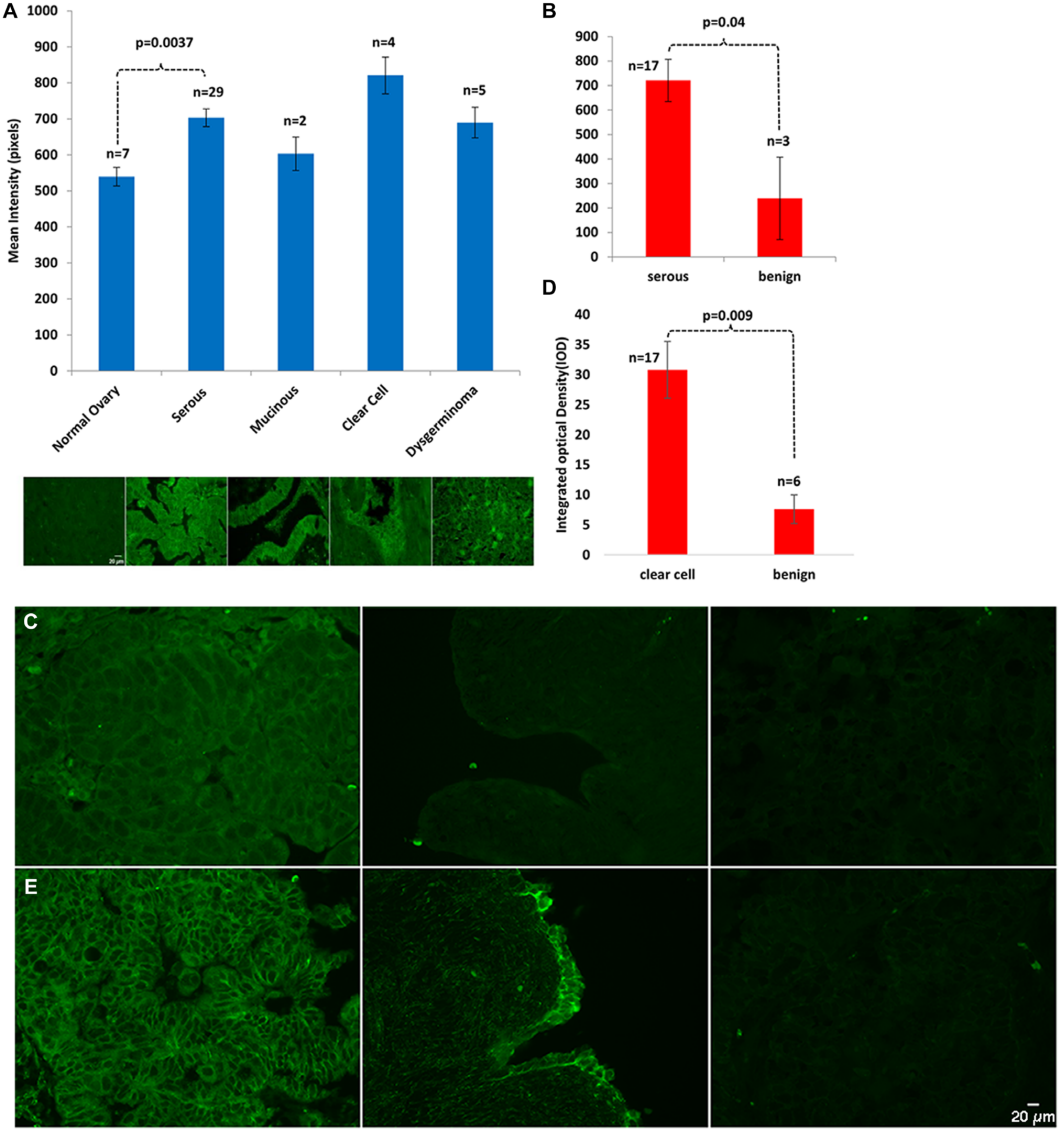
Septin-2 is overexpressed in EOC. (**A**) A commercially available human ovarian tissue microarray was stained with Septin-2 primary antibody and examined by confocal microscopy. Staining in all histopathogies was then quantified via mean intensity. (**B**) Paraffin-embedded human EOC tissue slides were stained for Septin-2 and expression was analyzed by integrated optical density. (**C**) Representative images of Serous EOC staining (left panel) vs benign (right panel). (**D**) Staining of paraffin-embedded human clear cell EOC tissue and benign controls with septin-2 was performed and expression was assessed by IOD. (**E**) Representative images of clear cell EOC staining (left panel) vs benign (right panel).

To establish the clinical relevance of septin-2 in EOC, we evaluated its levels in EOC samples of a variety of histologies, and compared these to levels in benign controls. Immunohistochemical analysis of septin-2 levels in a human ovarian tissue microarray comprising normal, serous, mucinous, clear cell, and dysgerminoma histopathologies revealed that mean intensity of the septin-2 staining was statistically significantly greater in serous EOC (703.38 pixels) than in adjacent normal tissue (539 pixels) (*p = 0.0037)* (Figure 1A). While all other histopatholgies exhibited higher mean intensity levels of septin-2—mucinous (603 pixels), clear cell (821 pixels), and dysgerminoma (744 pixels)—compared to the normal adjacent tissue, none were considered statistically significant, possibly due to low numbers of samples available.

To further investigate expression levels of septin-2 in patient samples, immunohistochemistry of septin-2 was performed in EOC and benign tissue from our institution. Integrated optical density (IOD) was calculated for each sample, which revealed statistically significant higher levels in serous (721 area*mean/1E+06, *p = 0.04)* (Figure 1B–1C) and clear cell (31 area*mean/1E+06, *p = 0.009)* histopathologies (Figure 1D–1E) compared to respective benign controls (239 area*mean/1E+06) and (6 area*mean/1E+06). Staining results were further validated with an independent antibody in five clear cell EOC, five serous EOC and four benign samples (Supplementary Figure 1A–1B).

### Stable knockdown of septin-2 influences cell proliferation

**Figure 2:**
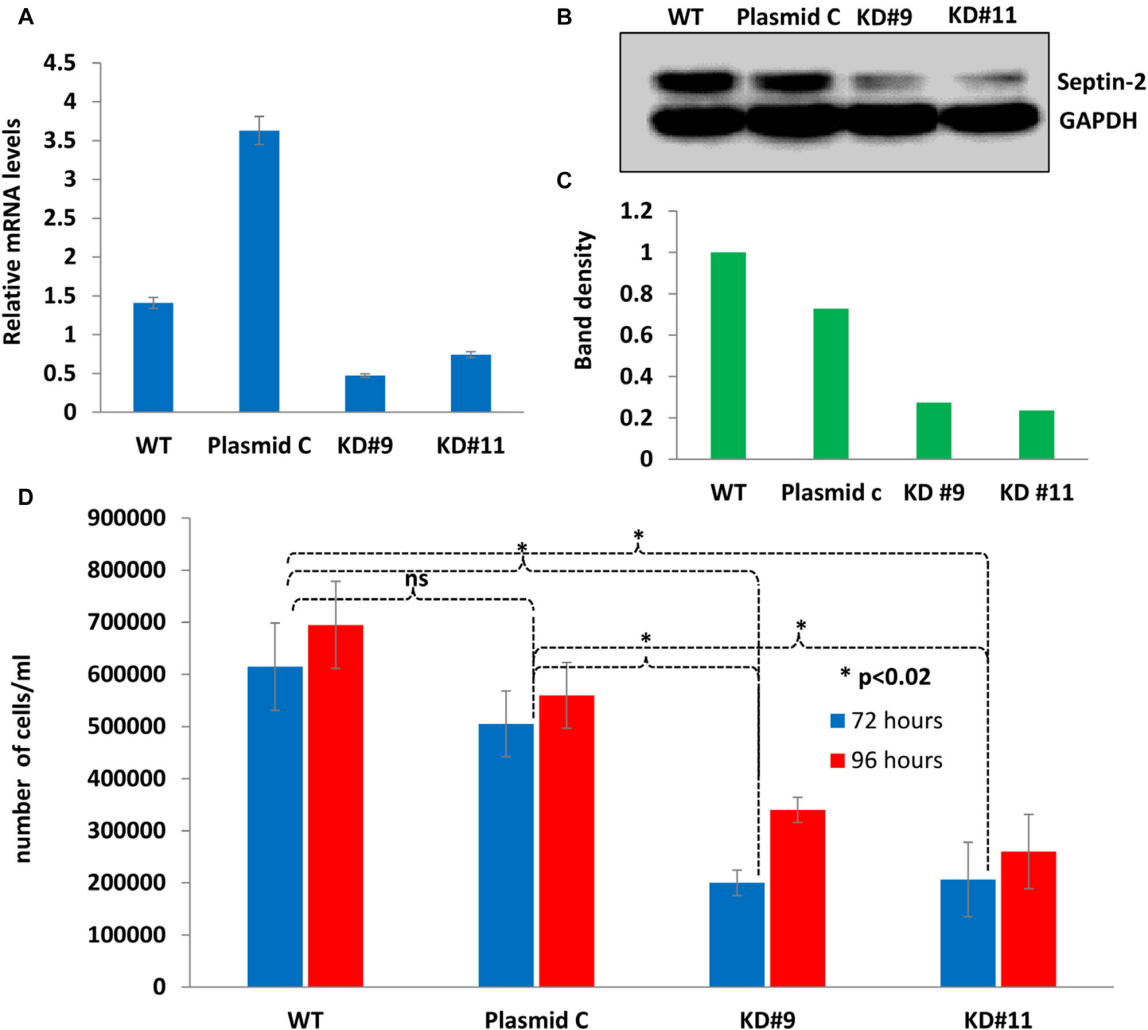
Stable septin-2 knockdown shows a decrease in proliferation. (**A**) Gene expression levels of septin-2 in KD9 and KD11 compared to WT and Plasmid C control levels determined by quantitative PCR (qPCR). (**B**) Verification of septin-2 knockdown at protein level visualized by Western blot. A single blot was probed simultaneously for septin-2 and GAPDH as a loading control. The original blot can be seen in Supplementary Figure 4. (**C**) Relative band density of (B) normalized to GAPDH. (**D**) Proliferation rates of KD9 and KD11 compared to control WT and Plasmid C were assessed at 72 and 96 hours by total live cell counts.

In order to study septin-2’s function in EOC, stable septin-2 knockdown shRNA clones were generated in human serous ovarian SKOV3 wild type (WT) cells. Two clonal populations were employed for these studies—knockdown 9 (KD9) and knockdown 11 (KD11)—based on confirmation of successful septin-2 downregulation. A stable line was also generated by clonal expansion of cells transfected with scrambled oligo control shRNA, designated Plasmid C. To confirm the efficacy of knockdowns at the genomic level, qPCR was employed. Septin-2 levels in KD9 were 1.93- and 4.16-fold lower than WT and Plasmid C cells, respectively. Septin-2 levels in KD11 were 1.67- and 3.88-fold lower than WT and Plasmid C cells, respectively (Figure 2A).

To further validate successful knockdown of septin-2, protein levels were detected by Western blot. We observed substantial decreases in septin-2 levels in KD9 and KD11 compared to the WT and Plasmid C controls (Figure 2B). Septin-2 levels in KD9 were 0.28-fold relative to WT and 0.38-fold reduced relative to Plasmid C. Septin-2 levels in KD11 were 0.24-fold and 0.32-fold reduced relative to WT and Plasmid C, respectively (Figure 2C).

To begin to determine the consequence of septin-2 knockdown in SKOV3 cells, proliferation of the shRNA clones was evaluated. WT, Plasmid C, KD9, and KD11 cells were seeded at equal cell densities and allowed to expand. The cells were trypsinized at 72 and 96 hours, and numbers of live cells in each clonal population were quantified (Figure 2D). At 72 hours, KD9 clones exhibited a 0.33-fold reduction in cell proliferation compared to WT, and a 0.40-fold reduction compared to Plasmid C. KD11 clones demonstrated a 0.34-fold and 0.41-fold reduction in proliferation from respective WT and Plasmid C cell numbers. The 96-hour time point revealed a 0.49-fold reduction in KD9 cells compared to WT and a 0.61-fold reduction compared to Plasmid C. KD11 cells showed a 0.37-fold and 0.46 fold- decrease compared to WT and Plasmid C cells, respectively. All decreases in cell counts displayed by KD9 and KD11 at both time points were determined to be statistically significant (*p < 0.02*). Differences in viability between WT, Plasmid C, KD9, and KD11 determined by live/dead counts were found to be non-significant (Supplementary Figure 2), confirming that our findings were not due to increased cell death. This finding strongly suggests that the downregulation of septin-2 has a profound impact on cell proliferation in EOC cells.

### Proteomic analysis of septin-2 knockdown in EOC cells

**Figure 3:**
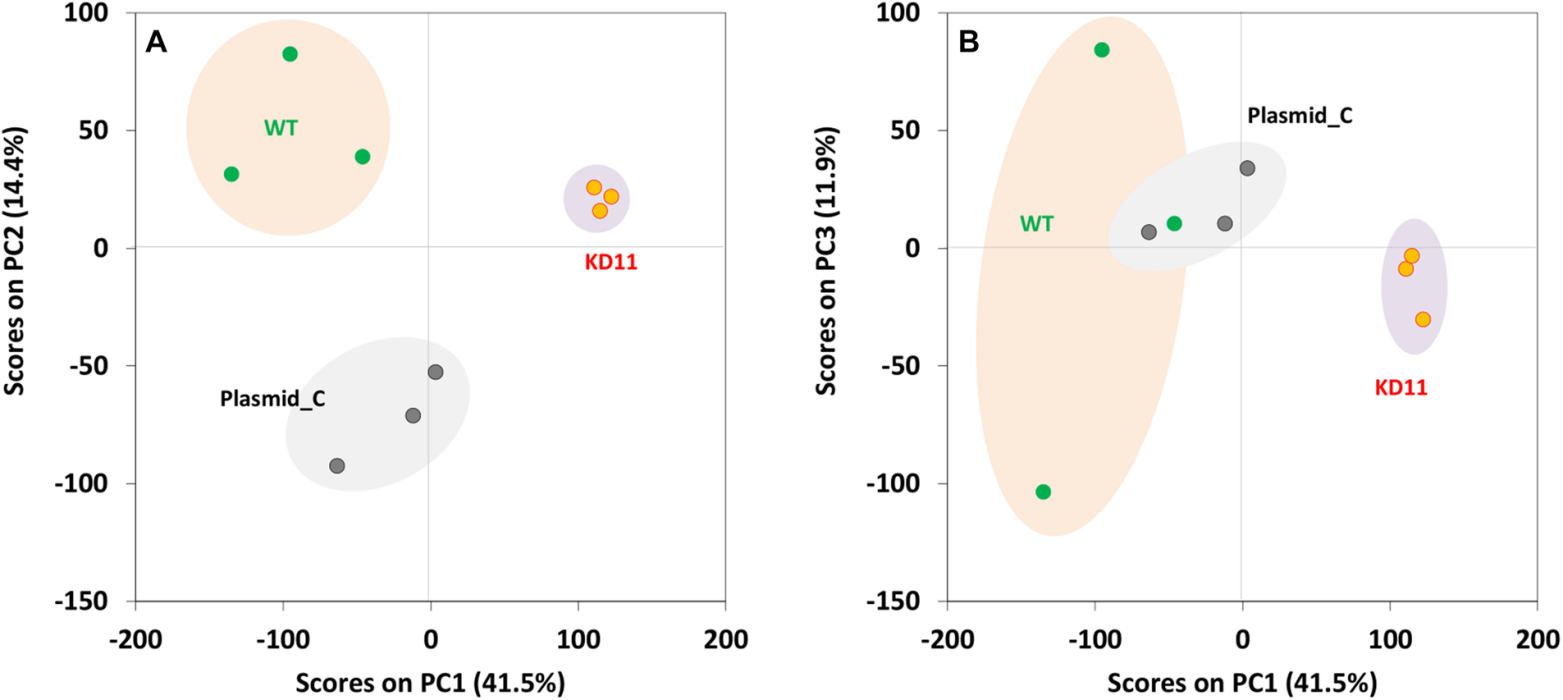
Principal component analysis of WT, Plasmid C, and KD11 samples. WT, Plasmid C and KD11 show clustering in visualization of principal component 1(PC1) versus principal component 2 (PC2) (**A**) wherein WT is more dispersed and shows overlap with Plasmid C in PC1 vs PC3 (**B**).

A comparative label-free proteomic analysis was performed to examine global protein expression level differences resulting from the knockdown of septin-2. Interestingly, significant differences in protein-peptide levels between control cells and septin-2 knockdowns was observed only in KD11 populations, even though our proliferation results demonstrated that KD9’s phenotype was similar to that of KD (Supplementary Dataset). In order to test the hypothesis that spontaneous loss of the knockdown had occurred in KD9 cells during cell culture, we reexamined septin-2 levels in each of the clonal cell lines and verified that septin-2 knockdown was indeed lost in KD9 cells, while the KD11 cells had retained septin-2 knockdown (Supplementary Figure 3). Therefore, we proceeded with analysis using KD11 cells. As expected, a principal component analysis of three biological replicates of WT, Plasmid C, and KD11 revealed separate clusters when comparing principal component 1 (PC1) and PC2 scores (Figure 3). In contrast, for KD9 sample, the 3 biological replicates were very scattered (data not shown). Therefore, any further analysis or validation of KD9 was not included.

**Figure 4:**
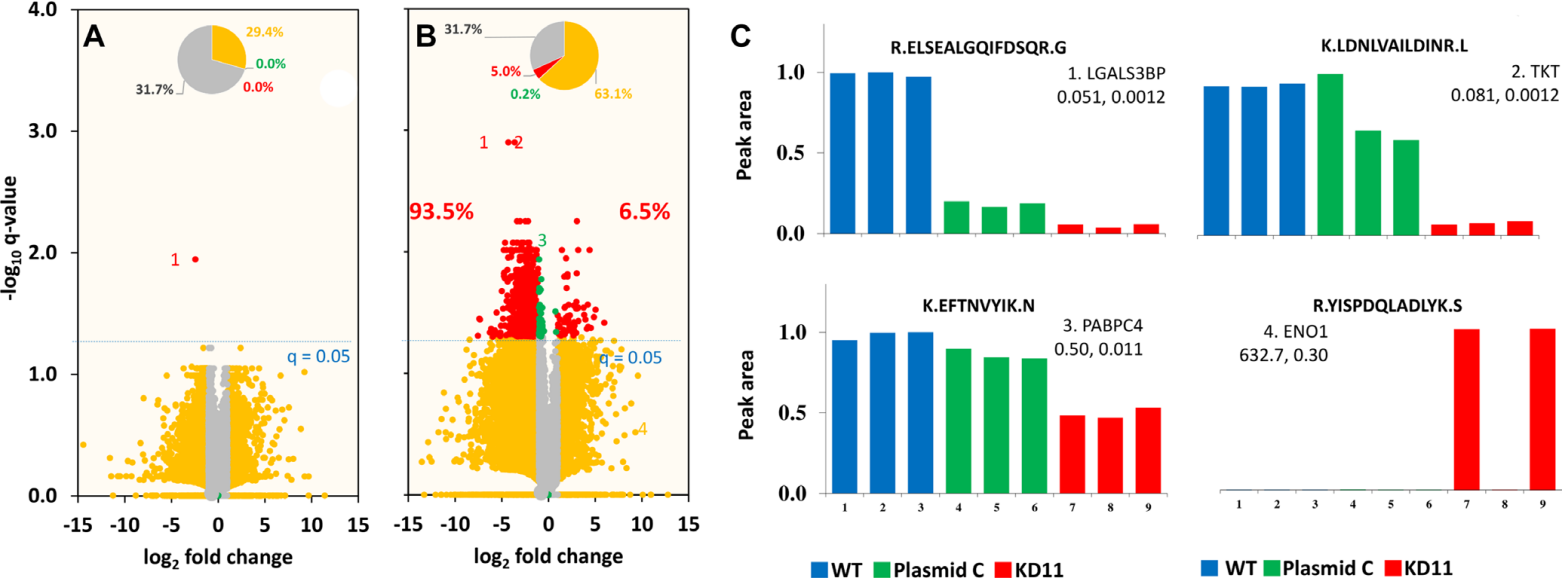
Volcano plot of fold change versus *q*-value of peak area for distinct peptides. Of the 19976 distinct peptides (3565 proteins) identified, (**A**) only one peptide/protein (0%, red in inset pie chart) in Plasmid C and (**B**) 5.0% peptides in KD11 showed large difference (absolute fold change more than 1, and *q* < 0.05) against WT. Nearly 93.5% peptides showed lower peak-area (down regulation) in KD11. (**C**) Examples of peak-area/expression levels in replicates for four peptides are shown: 1. Galectin-3-binding protein (LGALS3BP, UniProtID: K7EKQ5), 2. Transketolase (TKT, P29401), 3. Poly(A) binding protein 4 (PABPC4, Q4VC03), 4. Enolase 1 (ENO1, P06733). The peptide sequence, KD11/WT peak-area ratio, and respective *q*-values are listed for each protein.

**Figure 5:**
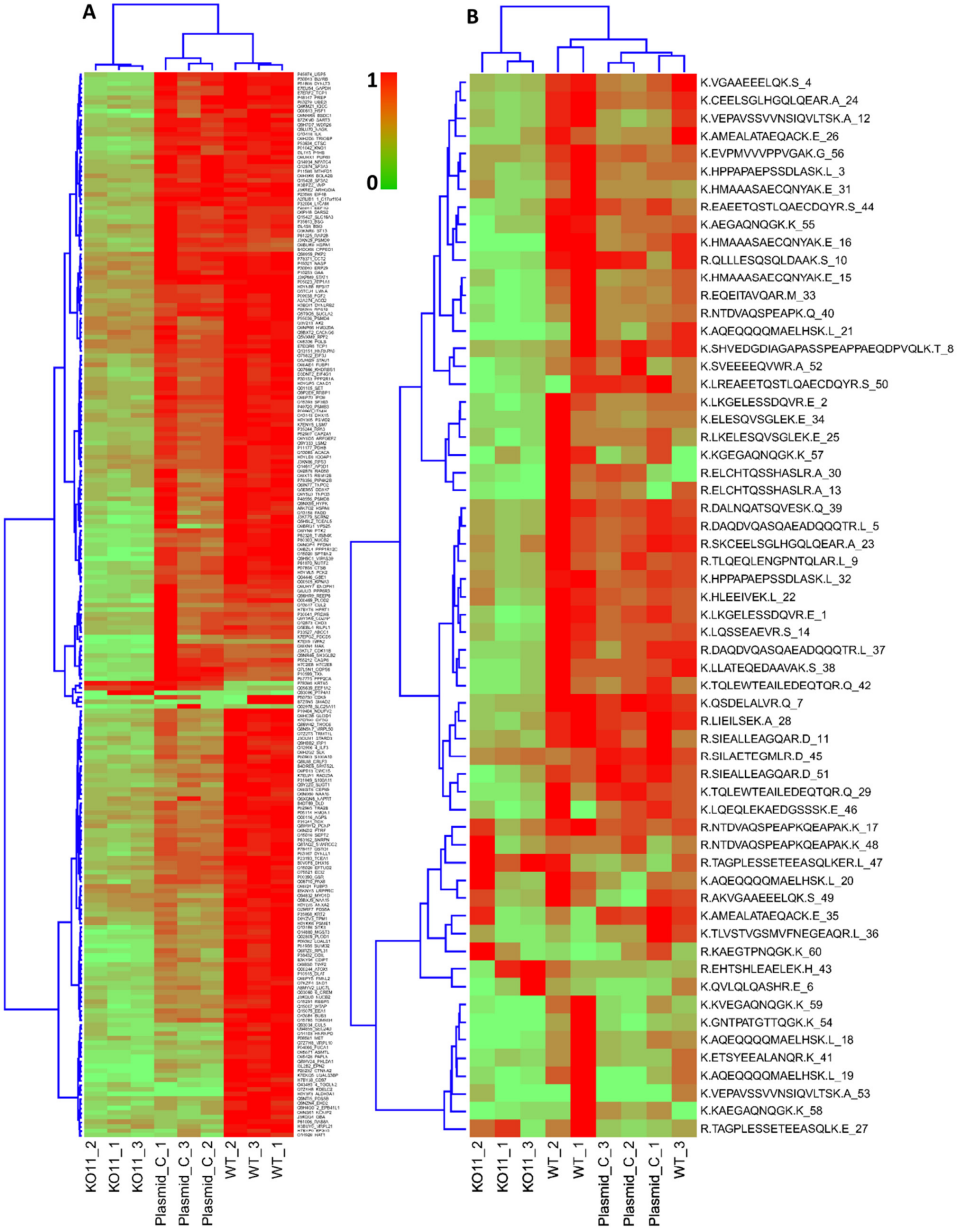
Hierarchical clustering and heat map of differentially expressed proteins and peptides. (**A**) Clustering of the 231 differentially expressed proteins. (**B**) Peptides in most differentially expressed proteins, for example, in ribosome binding protein 1(Q9P2E9 (RRBP1, Ribosome binding protein 1 UniProtID: Q9P2E9) with 60 peptides, showing an overall similar pattern of peak-area quantitation. Peptides/proteins show mostly down regulation in KD11 samples, which form a distinct cluster.

Mass spectrometry of the control and knockdown cells identified 19976 unique peptides corresponding to 3565 unique proteins. Of those, only one peptide/protein in Plasmid C exhibited an absolute fold change greater than 1 with a *q*-value < 0.05 compared to WT (Figure 4A). This result allowed us to conclude that there was no significant difference between both control cell populations. Conversely, 5% of all peptides in KD11 cells revealed relative fold change greater than 1 (*q* < 0.05) compared to WT cells. In addition, 93.5% of those peptides identified as exhibiting substantial expression differences displayed a lower peak area in KD11 compared to WT, indicating a majority of peptides were downregulated (Figure 4B). Representative examples of peak-area of four peptide sequences from the proteins galetin-3 binding protein (LFALS3BP), transketolase (TKT), poly(A) binding protein (PABPC4), and enolase-1(ENO1) show differential expression between control and knockdown cells. KD11/WT peak area ratios were calculated for LFALS3BP (0.051, *q* = 0.012), TKT (0.081, *q* = 0.0012), PABPC4 (0.50, *q* = 0.011), and ENO1 (632.7, *q* = 0.30) (Figure 4C). It is interesting to note that, all four of these proteins have previously been shown to play a role in tumorigenesis [18–21]. Heat maps were constructed to illustrate the clustering of the 231 differentially expressed proteins in each of the three replicates of WT, Plasmid C, and KD11 (Figure 5A) and representative peptides in the most differentially expressed proteins (Figure 5B). Comparison of both heat maps reveals an overall similar pattern of peak-area quantitation, with many of the proteins and peptide sequences within KD11 exhibiting downregulation compared to WT and Plasmid C controls.

**Figure 6:**
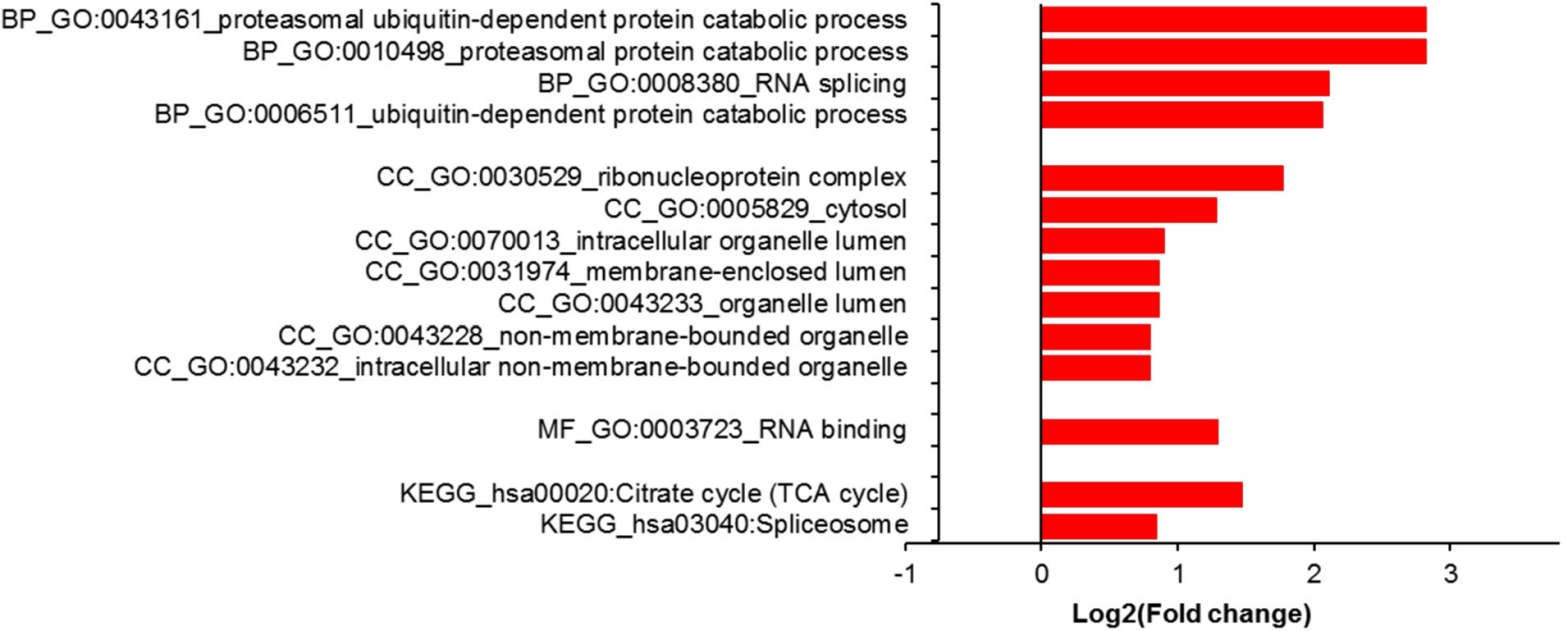
Gene ontology (GO) analysis using DAVID (https://david.ncifcrf.gov/). Proteins with differential expression (*n* = 231, *q* < 0.05, in KD11 versus WT is compared with proteins (*n* = 3334) that showed no differential expression. The former showed enrichment for proteasomal/ubiquitin related GO terms (*q* << 0.05, Bonferroni) in the biological process (BP) category. In cellular component (CC) and molecular function (MF) categories, differentially expressed proteins showed enrichment for ribonucleoprotein and RNA related terms. No enrichment was seen in the molecular function category. Differentially expressed proteins showed enrichment for KEGG pathways relating to citrate cycle/energy and spliceosome.

Finally, gene ontology (GO) analysis with differentially expressed proteins showed enrichment of proteasomal/ubiquitin in the biological process category and RNA binding in the molecular function category (Figure 6). Enrichment was also noted for terms related to the ribonucleoprotein complex and cytosol in the cellular component category. KEGG pathway analysis revealed citric acid cycle (TCA cycle) and spliceosome enrichment among differentially expressed proteins (Figure 6).

**Figure 7:**
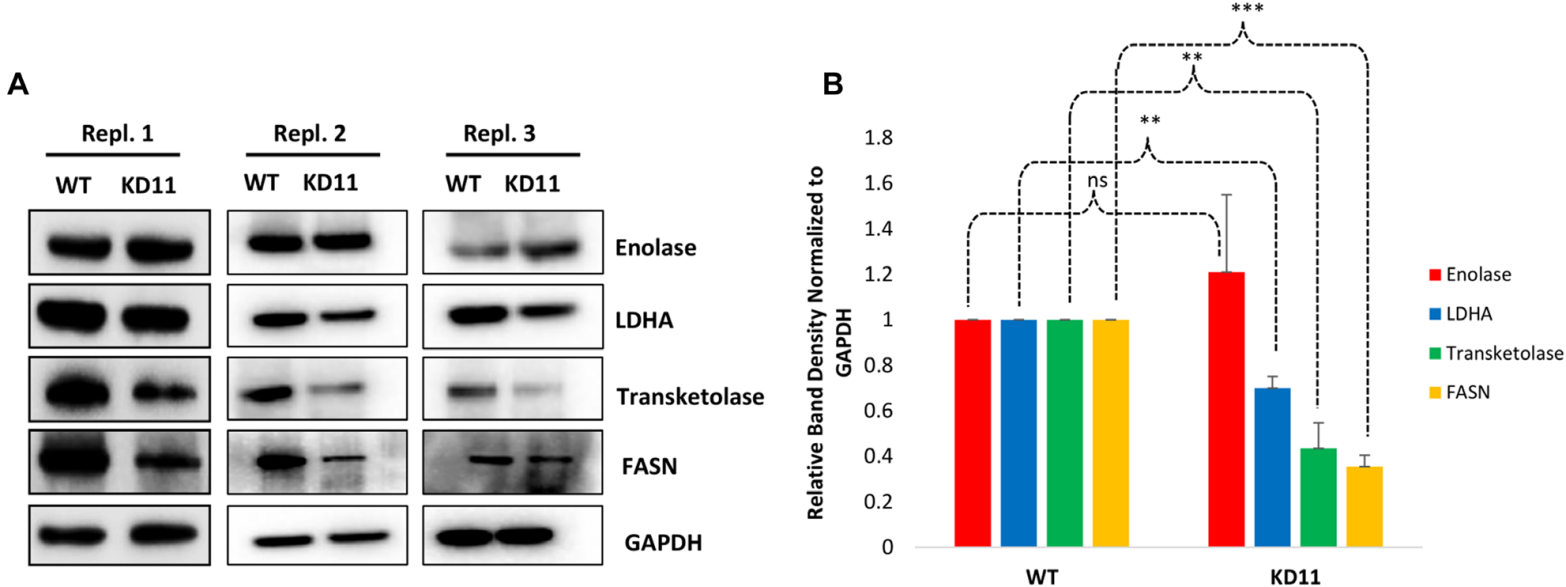
Verification of enriched proteins identified by proteomic analysis. (**A**) Triplicate Western blot analysis of protein expression validated in both WT and KD11. Blots were probed for GAPDH, then subsequently stripped and re-probed for each individual validated protein. Original blots from all three replicates can be seen in Supplementary Figure 4. (**B**) Relative Band Densities of proteins in (a), normalized to GAPDH. ^*^ *p <* 0.05 ^**^ *p <* 0.005, ^***^ *p <* 0.0005.

Representative proteins related to these pathways were further validated by immunoblot analysis in triplicate. Enolase, LDHA, Transketolase, and FASN expression in WT and KD11 was examined via Western blot (Figure 7A). Band density normalized to GAPDH revealed a 1.2-fold increase in Enolase expression from WT to KD11, although this change was not significant due to one replicate showing no increase. LDHA, Transketolase, and FASN levels in KD11 were 0.70-fold (*p* = 5.5 × 10^–4^), 0.43-fold (*p* = 0.001), and 0.35-fold (*p* = 2.5 × 10^–5^) lower relative to WT control. (Figure 7B).

## DISCUSSION

For the first time, we have characterized septin-2 function in EOC and examined its proteomic effects on a global level. Several biological pathways were found to be differentially regulated in septin-2 knockdown ovarian cancer cells, exemplified by representative proteins from Figure 4C. Galectin-3 is a member of the β-galactoside binding protein family that is involved in diverse functions inherent to cancer, such as metastasis, immune surveillance, inflammation, apoptosis, molecular trafficking, and mRNA splicing [22]. Transketolase is a pentose phosphate pathway enzyme essential for cancer growth due to its ability to control NADPH production and counteract oxidative stress [20]. Poly(A) binding protein is a highly conserved protein that plays an important role in mRNA stabilization and translation [23], which controls cell growth, proliferation, and differentiation [24]. Enolase1, found to be differentially expressed in cancer, is a key glycolytic enzyme that catalyzes 2-phosphogylgerate to phosphoenolpyruvate in the last steps of the glycolytic catabolic pathway [25].

Of these pathways identified, it was most expected that autophosphorylation and proteasomal/ubiquitin protein functions were affected by septin-2 knockdown. It has been previously established that proper control of septins’ phosphorylation status is required for the completion of cytokinesis [26]. In fungus, Meseroll et al (2013) discovered that changes in specific phosphorylation sites on septins (Cdc3p and Cdc11p) leads to the disruption of higher order septin structures, indicating septin phosphorylation is also a vital regulator of their own structure formation [27].

Similar to phosphorylation, ubiquitination represents another important septin post-translational modification. Septins have an established role interacting with proteins involved in degradation pathways, such as ubiquitin ligases and de-ubiquitylating enzymes, which modulates protein turnover [12, 28, 29]. Recently, it has also been reported that SUMOylation of human septins is a critical process contributing to proper septin filament bundling and cytokinesis [30]. Unlike ubiquitin, SUMO (small ubiquitin-like modifiers) modification does not always lead to protein degradation, as SUMOylation can also modulate localization, interaction, and activity of the target protein [31]. Ribet et.al (2017) reported that septin-7 is constitutively SUMOylated throughout the cell cycle, and septin variants that are unable to be SUMOylated halt septin bundle formation and lead to defects in cytokinesis, highlighting its crucial role in septin filament bundling and cell division [30].

GO analysis revealed that septin-2 is also involved in post-transcriptional modifications, as the spliceosome pathway was found to be enriched among septin-2 regulated proteins (Figure 6). This result suggests that septin-2 plays a major role in the editing of both precursor messenger RNA (pre-mRNA) and proteins. The spliceosome, a large molecular complex involved in the removal of non-coding introns from pre-mRNA, represents a potential oncogenic target as evidence has shown that tumors rely on normal spliceosome function for cell survival [32, 33]. In addition, Poly(A) binding protein, which we reported as an example of a differentially expressed protein (Figure 4C), is a translation initiation factor that binds to the mRNA 3’poly(A) tail [24] and influences cell growth and survival. Since we have shown that the knockdown of septin-2 promotes irregular expression of a multitude of pathways related to mRNA and protein modifications, it seems reasonable that its downregulation would also affect tumor cell growth.

As the depletion of septins can lead to cytokinesis failure, it is logical that cellular proliferation would subsequently be affected [30]. In this study, we observed a reduction in proliferation with septin-2 knockdown (Figure 2D). Corroborating results from our study in EOC, Zhang et al (2016) treated breast cancer cells with the broad septin inhibitor forchlorfenuron (FCF) and also observed a decrease in cell proliferation [17], which they attributed to the suppression of MEK and ERK1/2 (extracellular signal-regulated kinase 1/2) signaling [17]. Another study showed that septin-8 interacts with MAPK5 (mitogen activated protein kinase 5), further suggesting that septins play a role in the MAPK/ERK pathway [34]. Septin-9 has also been implicated in cell proliferation, as a septin-9 variant SEPT9_i1 binds to c-Jun-N terminal kinase (JNK), preventing its degradation and therefore promoting tumor cell proliferation [4]. In addition, another septin-9 variant SEPT9_i3 has been found to be phosphorylated by cell-cycle-dependent kinase 1 (CDK1), controlling entry into mitosis and promoting cell survival and proliferation [35]. These investigations highlight that septin-2, and septins in general, play an important role in cellular proliferation and potentially promote tumor growth.

Interestingly, the most novel conclusion drawn from this investigation was the robust enrichment seen in cellular metabolism and energy dynamics in proteins affected by septin-2 downregulation. This novel finding regarding septin-2 is in agreement with previous studies reporting on septin functions related to energy metabolism. One study found that in *Shigella* bacteria, host cell glycolysis becomes dysfunctional when septin-2 and septin-7 are depleted, indicating that septins may play a role in the modulation of glycolysis [36]. An additional study identified that fungal septins FaCdc3 and FaCdc12 are required for lipid metabolism [37]. Moreover, septin-9 was found to induce lipid droplet growth through binding to phosphatidylinositol-5-phosphate (PtdIns5P), a phospholipid with a well-established role in dynamics and intracellular membrane trafficking [38]. PtdIns5P binding in turn controls septin-9 filament formation and its interaction with microtubules [39]. Furthermore, septin-11 was found to be expressed in human adipocytes and upregulated in obese individuals. SEPT11 mRNA was positively correlated with markers of insulin resistance in adipose tissue, and silencing of septin-11 muted insulin signaling and insulin-induced lipid accumulation in adipocytes [40].

Our findings, however, represent the first time a septin family member has been implicated in cellular metabolism as it relates to tumorigenesis. Acetyl-CoA, one of the pathways most differentially expressed by septin-2, is a key metabolic player that links glycolysis, fatty acid oxidation, ketogenesis, amino acid metabolism, the TCA cycle, and lipid synthesis [41]. In normoxic conditions, acetyl-CoA is derived from glucose. However, under hypoxic conditions like in cancer, acetyl-CoA has been to found to derive from acetate, suggesting that targeting the acetyl-CoA pathway in cancer could represent a viable treatment option [42]. The TCA cycle, another important metabolic pathway, was also deregulated in the septin-2 knockdown clones. While previous dogma stated that tumor cells do not utilize the TCA cycle for energy, it has now been found that some cancer cells with deregulated oncogenes and tumor suppressor genes actually do rely on the TCA cycle [43]. In addition, the metabolic proteins transketolase and enolase, which are involved in glycolysis and the pentose phosphate pathway, respectively, were found to be differentially expressed by septin-2 inhibition (Figure 4C), demonstrating that septin-2 is involved in various facets of cellular metabolism within EOC. Pathways related to metabolism and energy production have previously been found to contribute to EOC tumorigenesis, as it has been shown that glycolysis drives chemoresistance in EOC and that high levels of fatty acid synthase (FASN) contribute to tumor cell growth through the promotion of human epidermal growth factor [44, 45]. Therefore, it can be hypothesized that the inhibition of septin-2 would exhibit a therapeutic effect in EOC via suppression of tumor metabolic pathways.

Overall, our study demonstrates the novel finding that septin-2 is involved in EOC pathogenesis. This investigation represents a springboard for future studies to determine the efficacy of septin-2 inhibition, in addition to more clearly elucidating its diverse mechanistic pathways in EOC tumorigenesis. While our proteomics study was performed in a serous ovarian cancer cell line, it would be interesting to repeat the stable knockdown experiment in a clear cell EOC line, since septin-2 was also found to be overexpressed in this histopathology. Additionally, both *in vitro* and *in vivo* studies could be performed to confirm that inhibition of septin-2 affects cell viability and tumor growth in order to determine if targeting of septin-2 synergizes with platinum-based chemotherapeutics.

## MATERIALS AND METHODS

### Cell culture

SKOV3 wild type (SKOV3WT) cells were obtained from American Type Culture Collection (ATCC) and were cultured at low passage in Dulbecco’s Modified Eagle Medium supplemented with 10% fetal bovine serum and 1% penicillin/streptomycin in a humidified incubator at 37°C/5% CO_2_.

### Septin-2 silencing with shRNA

shRNA for human septin-2 (Santa Cruz Biotechnology, sc-40936-SH) or scrambled oligonucleotide control shRNA (Plasmid-C; Santa Cruz Biotechnology, sc-108066) was transfected into SKOV3WT cells using Lipofectomine^®^ 2000 (Invitrogen, 11668) following the manufacturer’s instructions. Individual single cells were selected by culturing under the pressure of 5 ug/mL of puromycin (Research products International, 58-58-2), and clonal populations were allowed to expand. Phenotypes of the clones were evaluated by Western blotting using anti Septin-2 antibody (Novus Biologicals, NBP1-85212).

### Proliferation assay

SKOV3WT, Plasmid C, and knockdown lines (KD9 and KD11) were plated at equal densities in 100 × 20 mm plates. Cells were trypsinized at 72 and 96 hours, and replicates of three were counted using a hemocytometer with trypan blue staining to compare proliferation rates. The experiment was repeated three times and error bars represent standard deviation. Statistical significance was determined by an unpaired, two-tailed Student *t*-test, where *p <* 0.05 was considered significant. To ensure all cells had comparable viabilities, WT, Plasmid C, KD9, and KD11 were plated at equal densities in 60 × 20 mm plates. Cells were trypsinized at 72 hours and replicates of three were assessed via trypan blue staining and live/dead counts using a Nexcelom Biosciences Auto T4 Cellometer.

### Immunohistochemistry and confocal immunofluorescent microscopy

Formalin-fixed paraffin embedded human ovarian tissue slides were obtained from the Women and Infants Pathology Department. The human ovarian tissue microarray was obtained from US Tissue Biomax (OV802a). Slides obtained from Women and Infants were baked at 65°C for two hours, and the microarray for 20 minutes. All slides were subsequently washed in xylene, 100% ethanol, 95% ethanol, 70% ethanol, deoxygenated water, and FTA Hemagglutination Buffer. Antigen retrieval was then performed using DAKO antigen retrieval solution (10x) (Agilent, S1699), heated to 95°C for 20 minutes. Slides were then blocked with 5% horse serum in FTA Hemagglutination Buffer and incubated overnight in primary Septin-2 antibody (Santa Cruz, sc-20408) at 4°C. Secondary antibody, Alexa Fluora 488-conjugated donkey anti-Goat IgG (H+L) (Thermo Fisher Scientific, A-11055) was then added to slides following incubation in the dark for one hour at room temperature. Slides were washed in between steps with FTA Hemagglutination Buffer and were cover-slipped with DAPI containing mounting medium (Vector Laboratories, H-1200). While this primary Septin-2 antibody has been discontinued, results were replicated in a separate staining experiment in which the overexpression of septin-2 in serous and clear cell histopathogies compared to benign controls was confirmed with a commercially available antibody (Thermo Fisher Scientific PA5-27517). This antibody was also used to perform confirmatory staining of WT and KD11 cells. Secondary antibody Dylight 488 anti-rabbit IgG (Vector Laboratories, DI-1488) was then used (Supplementary Figure 1).

Images were acquired using a Nikon E800 microscope (Nikon Inc. Mellville, NY, USA) and an RT3 SPOT camera (Diagnostic Instruments, Sterling Heights, MI, USA). Random sampling of ten fields was based on DAPI staining. Mean intensity (used as a measurement for the tissue microarray as all areas for tissue were uniform) or integrated optical density (IOD) expressed as area*mean/1E+07, was acquired using a 40X objective. IOD was reported with staining performed with tissue obtained from our institution, as the size of each sample was variable. Statistical significance was determined by an unpaired, two-tailed Student *t*-test, where *p <* 0.05 was considered significant.

### Western blot

Protein was extracted from cell pellets in Cell Lysis Buffer (Cell Signaling, 9803) with 1 mM of PMSF, according to the manufacturer’s protocol. The concentration of extracted proteins was determined by DC Protein Assay (Bio-Rad Laboratories, 5000116). Western blot analysis was performed by loading equal amounts of protein boiled at 70°C with Novex Sample Reducing Agent (Life Technologies, NP009) and NuPAGE LDS sample buffer (ThermoFisher Scientific, NP0007) into a 4–12% gradient NuPAGE Novex Bis-Tris gel (Life Technologies, NP0321BOX). The gel was then transferred using a semi-dry transfer to methanol-activated 0.2 μm PVDF membranes (Bio-Rad, 162-0177) at 0.12-0.24A for 80 minutes. Membranes were blocked in 5% milk in phosphate-buffered saline with 0.05% Tween 20 (PBS-T) for 30 minutes at room temperature. Finally, membranes were incubated in primary antibody diluted in 5% milk in PBS-T overnight at 4°C, and then in secondary antibody diluted in 5% milk in PBS-T for 1 hour at room temperature, with PBS-T washes in between. Amersham ECL Prime Western Blot Detection System (GE Healthcare, RPN2232) was employed for detection of the HRP-tagged secondary antibodies. The Biorad Chemidoc MP Imaging System was used to image all blots. GAPDH was used as a loading control. Antibodies and respective dilutions used are as follows:

GAPDH (Cell Signaling, 2118, [1:2000])

Septin-2 (Novus Biologicals, NBP1-85212, [1:500])

LDHA (Cell Signaling, 3582S, [1:1000])

FASN (Cell Signaling, 3180S, [1:1000])

Enolase (Santa Cruz Biotechnology, sc-100812 [1:500])

Transketolase (Santa Cruz Biotechnology, sc-390179) [1:500]).

### Quantitative PCR

RNA was extracted from cells by Trizol /LiCl precipitation. Total RNA (1000 ng) was then reverse transcribed into cDNA using the iScript cDNA Synthesis Kit (Bio-Rad, 1708890), following the manufacturer’s protocol. Quantitative PCR was performed in triplicate by loading 1 μl cDNA reaction, 1 μM forward and reverse validated Septin-2 primers (Origene HP232247), 10 μl SYBR Green (Applied Biosciences [ABI], 4367659) and 5 μl RNAse-free water to each well. Samples were run using the ABI 7500 Fast Real-Time PCR System. Data was then analyzed using the ΔΔCt method. All gene expression levels were normalized to 18s rRNA.

### Densitometry

Densitometry analysis of Western blots was performed using image J. Blot images were analyzed in 8-bit JPEG format, using the “analyze gel” function. Relative band densities were normalized to GAPDH loading control.

### Sample preparation for LC-MS/MS analysis

Cell pellets were suspended in lysis buffer (8 M urea, 1 mM sodium orthovanadate, 20 mM HEPES, 2.5 mM sodium pyrophosphate, 1 mM β-glycerophosphate, pH 8.0, 20 min, 4°C), sonicated and cleared by centrifugation (14 000 × g, 15 min, 4°C). Protein concentration was measured (Pierce BCA Protein Assay, Thermo Fisher Scientific, IL, USA) and a total of 100 μg of protein per sample was digested with trypsin. Tryptic peptides were desalted using C18 Sep-Pak plus cartridges (Waters, Milford, MA, USA) and were lyophilized for 48 hours to dryness. The dried eluted peptides were reconstituted in buffer A (0.1 M acetic acid) at a concentration of 1 μg/μl, and 5 μl was injected for each analysis.

The LC-MS/MS was performed on a fully automated proteomic technology platform [46, 47] that includes an Agilent 1200 Series Quaternary HPLC system (Agilent Technologies, Santa Clara, CA, USA) connected to a Q Exactive Plus mass spectrometer (Thermo Fisher Scientific, Waltham, MA, USA). The LC-MS/MS set up was used as described previously [48]. Briefly, the peptides were separated through a linear reversed-phase 90 minutes gradient from 0% to 40% buffer B (0.1 M acetic acid in acetonitrile) at a flow rate of 3 μl /min through a 3 μm 20 cm C18 column. The electrospray voltage of 2.0 kV was applied in a split flow configuration, and spectra were collected using a top-9 data-dependent method. Survey full scan MS spectra (m/z 400-1800) were acquired at a resolution of 70,000 with an AGC target value of 3 × 106 ions or a maximum ion injection time of 200 ms. The peptide fragmentation was performed via higher-energy collision dissociation with the energy set at 28 NCE. The MS/MS spectra were acquired at a resolution of 17,500, with a targeted value of 2 × 104 ions or a maximum integration time of 200 ms. The ion selection abundance threshold was set at 8.0 × 102 with charge state exclusion of unassigned and *z* = 1, or 6–8 ions and dynamic exclusion time of 30 seconds.

### Database search

Peptide spectrum matching of MS/MS spectra of each file was searched against a species-specific database (UniProt; downloaded 2/1/2015) using MASCOT v. 2.4 (Matrix Science, Ltd, London, UK). A concatenated database containing “target” and “decoy” sequences was employed to estimate the false discovery rate (FDR) [49]. Msconvert from ProteoWizard (v. 3.0.5047), using default parameters and with the MS2Deisotope filter on, was employed to create peak lists for Mascot. The Mascot database search was performed with the following parameters: trypsin enzyme cleavage specificity, 2 possible missed cleavages, 10 ppm mass tolerance for precursor ions, 20 mmu mass tolerance for fragment ions. Search parameters permitted variable modification of methionine oxidation (+15.9949 Da) and static modification of carbamidomethylation (+57.0215 Da) on cysteine. The resulting peptide spectrum matches (PSMs) were reduced to sets of unique PSMs by eliminating lower scoring duplicates. To provide high confidence, the Mascot results were filtered for Mowse Score (>20). Peptide assignments from the database search were filtered down to a 1% FDR by a logistic spectral score as previously described [49, 50]. The mass spectrometry proteomics data have been deposited to the ProteomeXchange Consortium via the PRIDE partner repository with the dataset identifier PXD010166. Raw data is also available within the Supplementary Dataset file of this manuscript.

### Relative quantitation of the identified peptides

Relative quantification of peptide abundance was performed via calculation of selected ion chromatograms (SIC) peak areas. Retention time alignment of individual replicate analyses was performed as previously described [51]. Peak areas were calculated by inspection of SICs using in-house software programmed in R 3.0 based on the Scripps Center for Metabolomics’ XCMS package (version 1.40.0). This approach performed multiple passes through XCMS’ central wavelet transformation algorithm (implemented in the centWave function) over increasingly narrower ranges of peak widths, and used the following parameters: mass window of 10 ppm, minimum peak widths ranging from 2 to 20 seconds, maximum peak width of 80 seconds, signal to noise threshold of 10 and detection of peak limits via descent on the non-transformed data enabled. SIC peak areas were determined for every peptide that was identified by MS/MS. In the case of a missing MS/MS for a particular peptide, in a particular replicate, the SIC peak area was calculated according to the peptide’s isolated mass and the retention time calculated from retention time alignment. A minimum SIC peak area equivalent to the typical spectral noise level of 1000 was required of all data reported for label-free quantitation. Individual SIC peak areas were normalized to the peak area of the standard synthetic peptide DRVYHPF that was exogenously spiked prior to reversed-phase elution into the mass spectrometer. Quantitative analysis was applied to replicate experiments.

### Bioinformatics analyses

To select peptides that showed a statistically significant change in abundance between control vs treatment cells, *q*-values for multiple hypothesis tests were calculated based on *p*-values from two-tailed unpaired Student’s *t* tests using the R package QVALUE as previously described [52, 53]. Peptide peak-areas are summed to get protein-level quantification where required [54]. All calculations were done in R programming language and volcano plot/graphs were plotted in Excel.

Principal component analysis (PCA) and hierarchical clustering/heat map were also performed in R. Gene ontology (GO) analysis was performed using Enrichr (http://amp.pharm.mssm.edu/Enrichr/) for differentially expressed peptides/proteins [54] in KD11 versus WT against the background of unchanged proteins.

## SUPPLEMENTARY MATERIALS





## Abbreviations

EOC: epithelial ovarian cancer
GO: gene ontology
IOD: integrated optical density
KD: knockdown
PCA: principal component analysis
SIC: selected ion chromatograms

## References

[1] Dos Santos Guimarães I, Ladislau-Magescky T, Tessarollo NG, Dos Santos DZ, Gimba ERP, Sternberg C, Silva IV, Rangel LBA. Chemosensitizing effects of metformin on cisplatin- and paclitaxel-resistant ovarian cancer cell lines. Pharmacol Rep. 2018; 70:409–417. 10.1016/j.pharep.2017.11.007. 29627688

[2] Cancer Facts and Figures. American Cancer Society. 2018:1–77.

[3] Westin SN, Herzog TJ, Coleman RL. Investigational agents in development for the treatment of ovarian cancer. Invest New Drugs. 2013; 31:213–29. 10.1007/s10637-012-9837-3. 22661305 PMC4103697

[4] Angelis D, Spiliotis ET. Septin Mutations in Human Cancers. Front Cell Dev Biol. 2016; 4:122 10.3389/fcell.2016.00122. 27882315 PMC5101219

[5] Schmidt K, Nichols BJ. Functional interdependence between septin and actin cytoskeleton. BMC Cell Biol. 2004; 5:43 10.1186/1471-2121-5-43. 15541171 PMC535351

[6] Poüs C, Klipfel L, Baillet A. Cancer-related functions and subcellular localizations of septins. Front Cell Dev Biol. 2016; 4:126 10.3389/FCELL.2016.00126. 27878118 PMC5099157

[7] Kinoshita A, Kinoshita M, Akiyama H, Tomimoto H, Akiguchi I, Kumar S, Noda M, Kimura J. Identification of septins in neurofibrillary tangles in Alzheimer’s disease. Am J Pathol. 1998; 153:1551–60. 10.1016/S0002-9440(10)65743-4. 9811347 PMC1853406

[8] Saarikangas J, Barral Y. The emerging functions of septins in metazoans. EMBO Rep. 2011; 12:1118–26. 10.1038/embor.2011.193. 21997296 PMC3207108

[9] Russell SE, Hall PA. Do septins have a role in cancer? Br J Cancer. 2005; 93:499–503. 10.1038/sj.bjc.6602753. 16136025 PMC2361591

[10] Zhang M, He Y, Zhang X, Zhang M, Kong L. A pooled analysis of the diagnostic efficacy of plasmic methylated septin-9 as a novel biomarker for colorectal cancer. Biomed Rep. 2017; 7:353–360. 10.3892/br.2017.970. 29085631 PMC5649537

[11] Yu J, Zhang W, Tang H, Qian H, Yang J, Zhu Z, Ren P, Lu B. Septin 2 accelerates the progression of biliary tract cancer and is negatively regulated by mir-140-5p. Gene. 2016; 589:20–6. 10.1016/j.gene.2016.05.005. 27155525

[12] Marcus EA, Tokhtaeva E, Turdikulova S, Capri J, Whitelegge JP, Scott DR, Sachs G, Berditchevski F, Vagin O. Septin oligomerization regulates persistent expression of ErbB2/HER2 in gastric cancer cells. Biochem J. 2016; 473:1703–18. 10.1042/BCJ20160203. 27048593 PMC4903893

[13] Jian W, Zhong L, Wen J, Tang Y, Qiu B, Wu Z, Yan J, Zhou X, Zhao T. SEPTIN2 and STATHMIN regulate CD99-mediated cellular differentiation in Hodgkin’s lymphoma. PLoS One. 2015; 10:e0127568 10.1371/journal.pone.0127568. 26000982 PMC4441373

[14] Cao LQ, Shao ZL, Liang HH, Zhang DW, Yang XW, Jiang XF, Xue P. Activation of peroxisome proliferator-activated receptor-γ (PPARγ) inhibits hepatoma cell growth via downregulation of SEPT2 expression. Cancer Lett. 2015; 359:127–35. 10.1016/j.canlet.2015.01.004. 25592041

[15] Craven RA, Stanley AJ, Hanrahan S, Dods J, Unwin R, Totty N, Harnden P, Eardley I, Selby PJ, Banks RE. Proteomic analysis of primary cell lines identifies protein changes present in renal cell carcinoma. Proteomics. 2006; 6:2853–64. 10.1002/pmic.200500549. 16596713

[16] Craven RA, Hanrahan S, Totty N, Harnden P, Stanley AJ, Maher ER, Harris AL, Trimble WS, Selby PJ, Banks RE. Proteomic identification of a role for the von Hippel Lindau tumour suppressor in changes in the expression of mitochondrial proteins and septin 2 in renal cell carcinoma. Proteomics. 2006; 6:3880–93. 10.1002/pmic.200500811. 16739133

[17] Zhang N, Liu L, Fan N, Zhang Q, Wang W, Zheng M, Ma L, Li Y, Shi L. The requirement of SEPT2 and SEPT7 for migration and invasion in human breast cancer via MEK/ERK activation. Oncotarget. 2016; 7:61587–600. 10.18632/oncotarget.11402. 27557506 PMC5308674

[18] Song L, Tang JW, Owusu L, Sun MZ, Wu J, Zhang J. Galectin-3 in cancer. Clin Chim Acta. 2014; 431:185–91. 24530298 10.1016/j.cca.2014.01.019

[19] Liu D, Yin B, Wang Q, Ju W, Chen Y, Qiu H, Li J, Peng X, Lu C. Cytoplasmic poly(A) binding protein 4 is highly expressed in human colorectal cancer and correlates with better prognosis. J Genet Genomics. 2012; 39:369–74. 10.1016/j.jgg.2012.05.007. 22884093

[20] Xu IM, Lai RK, Lin SH, Tse AP, Chiu DK, Koh HY, Law CT, Wong CM, Cai Z, Wong CC, Ng IO. Transketolase counteracts oxidative stress to drive cancer development. Proc Natl Acad Sci U S A. 2016; 113:E725–34. 26811478 10.1073/pnas.1508779113 PMC4760787

[21] Qian X, Xu W, Xu J, Shi Q, Li J, Weng Y, Jiang Z, Feng L, Wang X, Zhou J, Jin H. Enolase 1 stimulates glycolysis to promote chemoresistance in gastric cancer. Oncotarget. 2017; 8:47691–708. 10.18632/oncotarget.17868. 28548950 PMC5564598

[22] Ruvolo PP. Galectin 3 as a guardian of the tumor microenvironment. Biochim Biophys Acta. 2016; 1863:427–437. 26264495 10.1016/j.bbamcr.2015.08.008

[23] Dizin E, Gressier C, Magnard C, Ray H, Didier D, Ohlmann T, Dalla Venezia D. BRCA1 Interacts with Poly(A)-binding Protein implication of brca1 in translation regulation. J Biol Chem. 2006; 281:24236–46. 16782705 10.1074/jbc.M602176200

[24] Yoshida M, Yoshida K, Kozlov G, Lim NS, De Crescenzo G, Pang Z, Berlanga JJ, Kahvejian A, Gehring K, Wing SS, Sonenberg N. Poly(A) binding protein (PABP) homeostasis is mediated by the stability of its inhibitor, Paip2. EMBO J. 2006; 25:1934–44. 10.1038/sj.emboj.7601079. 16601676 PMC1456944

[25] Díaz-Ramos À, Roig-Borrellas A, García-Melero A, López-Alemany R. α-enolase, a multifunctional protein:Its role on pathophysiological situations. J Biomed Biotechnol. 2012; 2012:156795 10.1155/2012/156795. 23118496 PMC3479624

[26] Dobbelaere J, Gentry MS, Hallberg RL, Barral Y. Phosphorylation-dependent regulation of septin dynamics during the cell cycle. Dev Cell. 2003; 4:345–57. 10.1016/S1534-5807(03)00061-3. 12636916

[27] Meseroll RA, Occhipinti P, Gladfelter AS. Septin phosphorylation and coiled-coil domains function in cell and septin ring morphology in the filamentous fungus Ashbya gossypii. Eukaryot Cell. 2013; 12:182–93. 10.1128/EC.00251-12. 23204191 PMC3571309

[28] Nakahira M, Macedo JN, Seraphim TV, Cavalcante N, Souza TA, Damalio JC, Reyes LF, Assmann EM, Alborghetti MR, Garratt RC, Araujo AP, Zanchin NI, Barbosa JA, Kobarg J. A draft of the human septin interactome. PLoS One. 2010; 5:e13799 10.1371/journal.pone.0013799. 21082023 PMC2970546

[29] Diesenberg K, Beerbaum M, Fink U, Schmieder P, Krauss M. SEPT9 negatively regulates ubiquitin-dependent downregulation of EGFR. J Cell Sci. 2015; 128:397–407. 10.1242/jcs.162206. 25472714

[30] Ribet D, Boscaini S, Cauvin C, Siguier M, Mostowy S, Echard A, Cossart P. SUMOylation of human septins is critical for septin filament bundling and cytokinesis. J Cell Biol. 2017; 216:4041–52. 10.1083/jcb.201703096. 29051266 PMC5716278

[31] Westerbeck JW, Pasupala N, Guillotte M, Szymanski E, Matson BC, Esteban C, Kerscher O. A SUMO-targeted ubiquitin ligase is involved in the degradation of the nuclear pool of the SUMO E3 ligase Siz1. Mol Biol Cell. 2014; 25:1–16. 10.1091/mbc.E13-05-0291. 24196836 PMC3873881

[32] van Alphen RJ, Wiemer EA, Burger H, Eskens FA. The spliceosome as target for anticancer treatment. Br J Cancer. 2009; 100:228–32. 10.1038/sj.bjc.6604801. 19034274 PMC2634708

[33] Lee SC, Abdel-Wahab O. Therapeutic targeting of splicing in cancer. Nat Med. 2016; 22:976–86. 10.1038/nm.4165. 27603132 PMC5644489

[34] Shiryaev A, Kostenko S, Dumitriu G, Moens U. Septin 8 is an interaction partner and *in vitro* substrate of MK5. World J Biol Chem. 2012; 3:98–109. 10.4331/wjbc.v3.i5.98. 22649572 PMC3362842

[35] Liu P, Kao TP, Huang H. CDK1 promotes cell proliferation and survival via phosphorylation and inhibition of FOXO1 transcription factor. Oncogene. 2008; 27:4733–44. 10.1038/onc.2008.104. 18408765

[36] Lobato-Márquez D, Krokowski S, Sirianni A, Larrouy-Maumus G, Mostowy S. A requirement for septins and the autophagy receptor p62 in the proliferation of intracellular Shigella. Cytoskeleton (Hoboken). 2018 5 12 10.1002/cm.21453. [Epub ahead of print]. PMC6519264 29752866

[37] Zhang Y, Gao T, Shao W, Zheng Z, Zhou M, Chen C. The septins FaCdc3 and FaCdc12 are required for cytokinesis and affect asexual and sexual development, lipid metabolism and virulence in Fusarium asiaticum. Mol Plant Pathol. 2017; 18:1282–94. 27666337 10.1111/mpp.12492 PMC6638246

[38] Di Paolo G, De Camilli P. Phosphoinositides in cell regulation and membrane dynamics. Nature. 2006; 443:651–7. 10.1038/nature05185. 17035995

[39] Akil A, Peng J, Omrane M, Gondeau C, Desterke C, Marin M, Tronchère H, Taveneau C, Sar S, Briolotti P, Benjelloun S, Benjouad A, Maurel P, et al Septin 9 induces lipid droplets growth by a phosphatidylinositol-5-phosphate and microtubule-dependent mechanism hijacked by HCV. Nat Commun. 2016; 7:12203 10.1038/ncomms12203. 27417143 PMC4947189

[40] Moreno-Castellanos N, Rodríguez A, Rabanal-Ruiz Y, Fernández-Vega A, López-Miranda J, Vázquez-Martínez R, Frühbeck G, Malagón MM. The cytoskeletal protein septin 11 is associated with human obesity and is involved in adipocyte lipid storage and metabolism. Diabetologia. 2017; 60:324–35. 10.1007/s00125-016-4155-5. 27866222

[41] Lee JV, Shah SA, Wellen KE. Obesity, cancer and acetyl-CoA metabolism. Drug Discov Today Dis Mech. 2013; 10:e55–e61. 10.1016/j.ddmec.2013.03.005. 23878588 PMC3713850

[42] Kamphorst JJ, Chung MK, Fan J, Rabinowitz JD. Quantitative analysis of acetyl-CoA production in hypoxic cancer cells reveals substantial contribution from acetate. Cancer Metab. 2014; 2:23 10.1186/2049-3002-2-23. 25671109 PMC4322440

[43] Anderson NM, Mucka P, Kern JG, Feng H. The emerging role and targetability of the TCA cycle in cancer metabolism. Protein Cell. 2018; 9:216–237. 10.1007/s13238-017-0451-1. 28748451 PMC5818369

[44] Chakraborty PK, Mustafi SB, Xiong X, Dwivedi SKD, Nesin V, Saha S, Zhang M, Dhanasekaran D, Jayaraman M, Mannel R, Moore K, McMeekin S, Yang D, et al MICU1 drives glycolysis and chemoresistance in ovarian cancer. Nat Commun. 2017; 8:14634 10.1038/ncomms14634. 28530221 PMC5477507

[45] Tania M, Khan MA, Song Y. Association of lipid metabolism with ovarian cancer. Curr Oncol. 2010; 17:6–11. 10.3747/co.v17i5.668. PMC2949373 20975872

[46] Yu K, Salomon AR. HTAPP: High-throughput autonomous proteomic pipeline. Proteomics. 2010; 10:2113–22. 10.1002/pmic.200900159. 20336676 PMC3214964

[47] Yu K, Salomon AR. PeptideDepot: Flexible relational database for visual analysis of quantitative proteomic data and integration of existing protein information. Proteomics. 2009; 9:5350–8. 10.1002/pmic.200900119. 19834895 PMC2831646

[48] Ahsan N, Belmont J, Chen Z, Clifton JG, Salomon AR. Highly reproducible improved label-free quantitative analysis of cellular phosphoproteome by optimization of LC-MS/MS gradient and analytical column construction. J Proteomics. 2017; 165:69–74. 10.1016/j.jprot.2017.06.013. 28634120 PMC5542054

[49] Elias JE, Gygi SP. Target-decoy search strategy for increased confidence in large-scale protein identifications by mass spectrometry. Nat Methods. 2007; 4:207–14. 10.1038/nmeth1019. 17327847

[50] Yu K, Sabelli A, DeKeukelaere L, Park R, Sindi S, Gatsonis CA, Salomon A. Integrated platform for manual and high-throughput statistical validation of tandem mass spectra. Proteomics. 2009; 9:3115–25. 10.1002/pmic.200800899. 19526561 PMC3122135

[51] Demirkan G, Yu K, Boylan JM, Salomon AR, Gruppuso PA. Phosphoproteomic profiling of *in vivo* signaling in liver by the mammalian target of rapamycin complex 1 (mTORC1). PLoS One. 2011; 6:e21729 10.1371/journal.pone.0021729. 21738781 PMC3125343

[52] Storey JD. The positive false discovery rate: A Bayesian interpretation and the *q*-value. Ann Stat. 2003; 31:2013–35. 10.1214/aos/1074290335.

[53] Storey JD, Tibshirani R. Statistical significance for genomewide studies. Proc Natl Acad Sci U S A. 2003; 100:9440–5. 10.1073/pnas.1530509100. 12883005 PMC170937

[54] Carrillo B, Yanofsky C, Laboissiere S, Nadon R, Kearney RE. Methods for combining peptide intensities to estimate relative protein abundance. Bioinformatics. 2009; 26:98–103. 10.1093/bioinformatics/btp610. 19892804

