# Supplementary material for: Septin-2 is overexpressed in epithelial ovarian cancer and mediates proliferation via regulation of cellular metabolic proteins
Source: Oncotarget. 2019 Apr 26;10(31):2959–72. doi: 10.18632/oncotarget.26836 (PMC6508204; doi:10.18632/oncotarget.26836)
Supplement: Supplementary file 1 [file oncotarget-10-2959-s001.pdf]

Septin-2 is overexpressed in epithelial ovarian cancer and mediates proliferation via regulation of cellular metabolic proteins

SUPPLEMENTARY MATERIALS

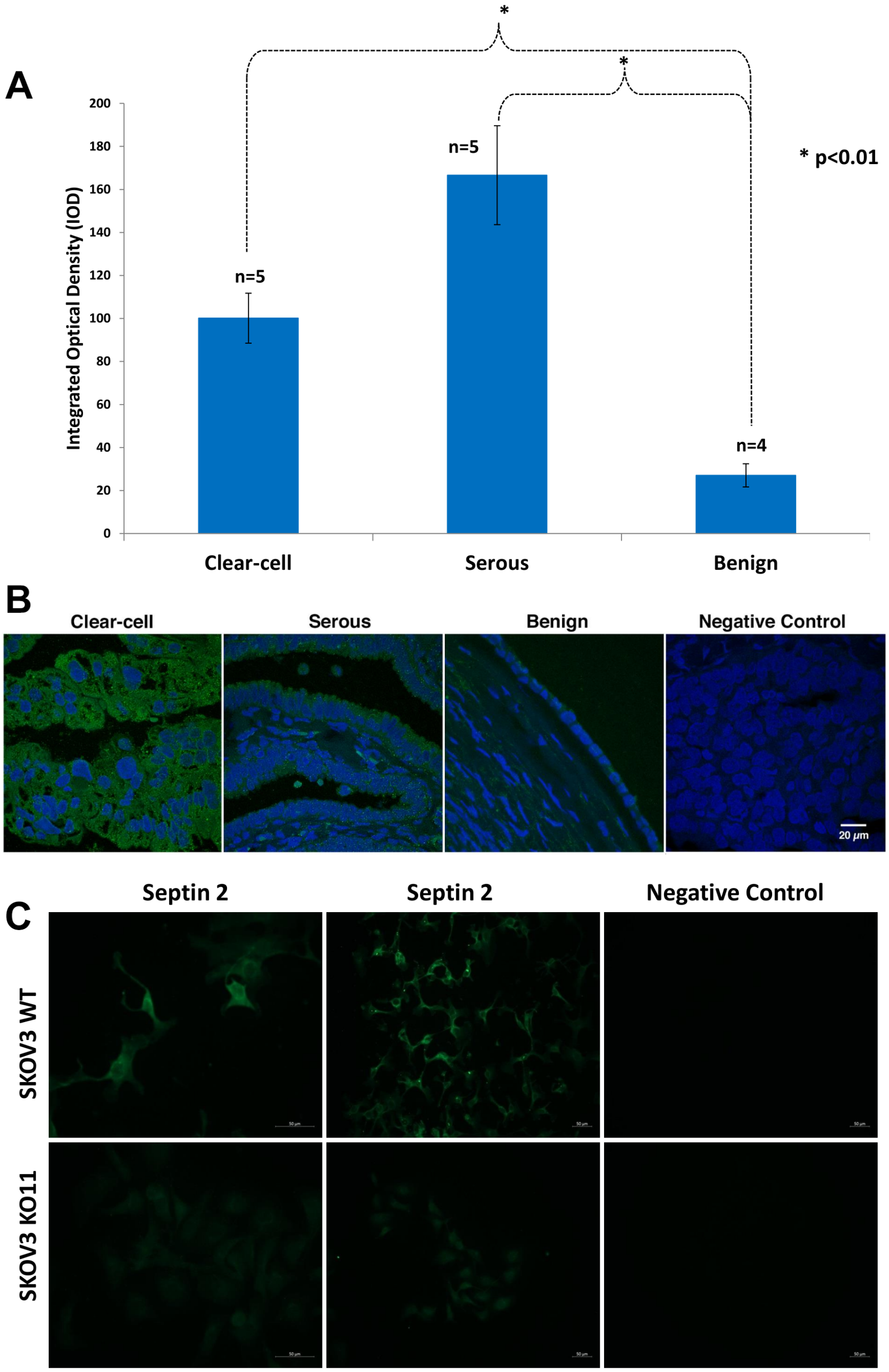

Supplementary Figure 1: Confirmation of Septin-2 overexpression in EOC. (**A**) Paraffin-embedded, serous, clear cell EOC and benign ovarian control tissue were stained using commercially available septin-2 antibody. Levels of septin-2 within each histopathogy were quantified by IOD. (**B**) Representative images of septin-2 staining in serous, clear cell and benign tissue. (**C**) Representative images of septin-2 staining in WT and KD11 cells, imaged at 40x and 20x magnification.

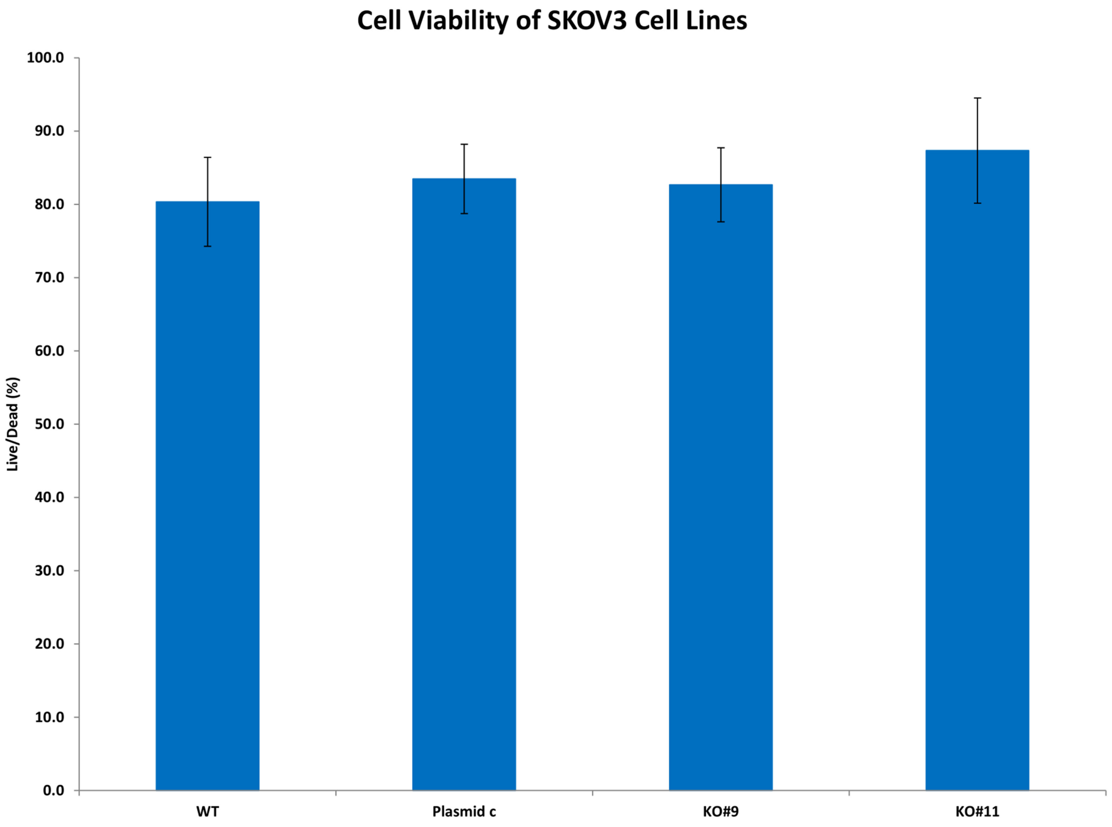

Supplementary Figure 2: Cell viability assessment of Septin-2 clones. Cell viability was assessed at 72 hours by live/dead counts, and percentages of viable cells across all control and clonal populations were found to be non-significant.

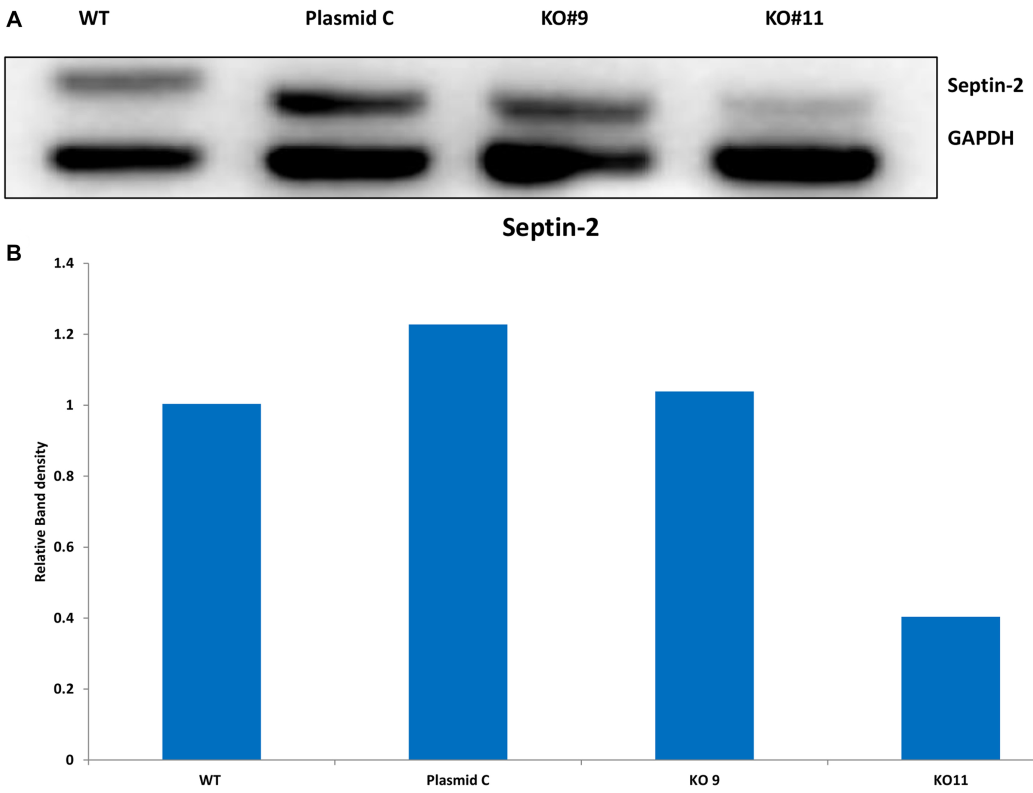

Supplementary Figure 3: Loss of Septin-2 knockdown in KD9 clones. (**A**) Western blot was performed to visualized septin-2 protein levels in WT, Plasmid C, KD9, and KD11 expression levels. A single blot was probed simultaneously for septin-2 and GAPDH as a loading control. The original blot can be seen in Supplementary Figure 4. (**B**) Relative band density of (a) normalized to GAPDH.

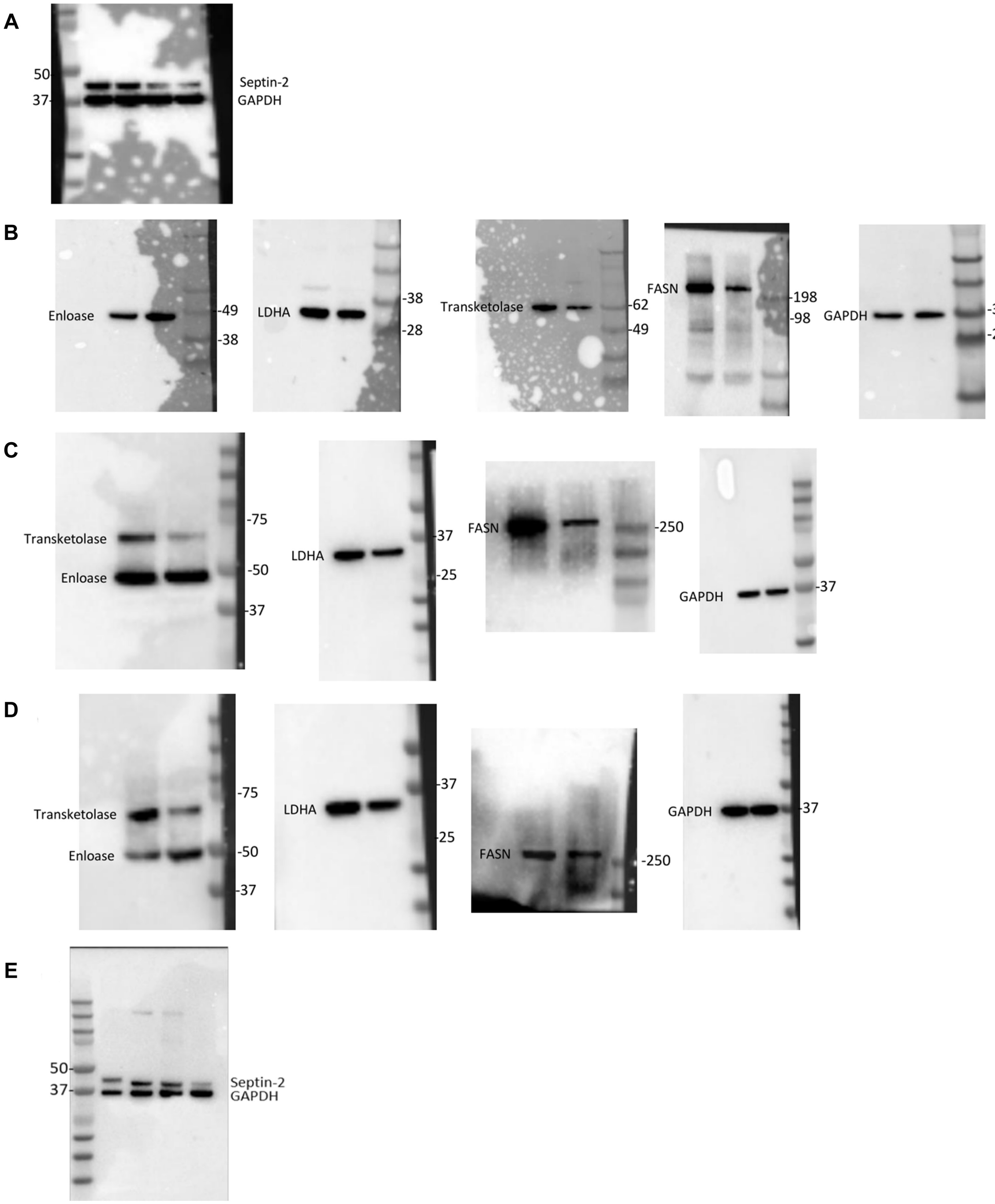

Supplementary Figure 4: Original uncropped Western blots. (**A**) Original blot from Figure 2B, merged with ladder image. (**B**–**D**) Original blots from Figure 7A, merged with ladder. (**E**) Original blot from Supplementary Figure 3, merged with ladder.

Supplementary Dataset: Complete proteomics data, including peak areas, ratios, *q*-values, and gene ontology annotations for all peptides. See Supplementary_Dataset.
